# Incidence and type of restrictive practice use in nursing homes in Ireland

**DOI:** 10.1186/s12877-022-03450-4

**Published:** 2022-10-15

**Authors:** Paul Dunbar, Martin McMahon, Ciara Durkan, Kieran A. Walsh, Laura M. Keyes

**Affiliations:** 1 Health Information and Quality Authority, Unit 1301, City Gate, Mahon, Cork, T12 Y2XT Ireland; 2 School of Medicine, University College Dublin, Dublin, Ireland

**Keywords:** Residential care facilities, Human rights, Person-centred care, Restraint

## Abstract

**Background:**

Use of restrictive practices (RP) in care settings may sometimes be warranted but can also conflict with human rights. Research to date has focused primarily on physical and chemical RP, however other forms are also used. Better understanding of practice can inform RP reduction. This study describes the incidence of all types of RP use reported from nursing homes in Ireland.

**Methods:**

RP notifications from nursing homes reported in 2020 were extracted from the Database of Statutory Notifications from Social Care in Ireland. The primary outcome measurement was the national incidence of use (frequency of RP/occupancy per 1000 residents) of categories and types of RP. Secondary outcome measurements such as percentage of facilities reporting use and quarterly median incidence of use in these facilities were calculated.

**Results:**

Seventy thousand six hundred sixty-three RP uses were notified from 608 facilities (33,219 beds). National incidence of RP use per 1000 residents was, all categories: 2465.1, environmental: 1324.5, physical: 922.5, chemical: 141.1; ‘other’: 77.0. The most frequently used RPs per category were, environmental: door locks; physical: bedrails; chemical (where drug specified): antipsychotics; ‘other’: privacy. 90.5% of nursing homes reported using at least one type of RP in the 12-month period. Quarterly incidence of any RP use in these facilities was median 1.642 (IQR: 0.018 to 18.608) per bed.

**Conclusions:**

Nursing homes in Ireland regularly use RP; only 9.5% reported no RP use in the 12-month period. A wide variety of types of RP were reported. Environmental and ‘other’ (largely psychosocial) RP contributed notably to total RP use and warrant attention alongside the traditional focus on physical and chemical RP. Policy implications include the need for more comprehensive RP definitions.

**Supplementary Information:**

The online version contains supplementary material available at 10.1186/s12877-022-03450-4.

## Background and objectives

While providing care in residential services for older adults (hereafter referred to as ‘nursing homes’) it may occasionally become necessary to use forms of restraint, restrictive interventions or restrictive practices (RP). These terms are often used interchangeably; for clarity, RP is used hereafter to encompass all above terms.

RP in the context of nursing homes can be defined as “activities or interventions, either physical or pharmacological, that have the effect of restricting a person’s free movement or ability to make decisions” [1]. The use of RP is increasingly seen as an infringement of human rights principles e.g. liberty, dignity and bodily integrity [2–4]. Care providers in many jurisdictions are actively encouraged to reduce use of RP; regulations or legislation often underpin this effort [5–7].

RP are typically broken down into categories such as physical, chemical, mechanical, environmental, seclusion, psychosocial or psychological [8, 9]. Research on RP use is complicated by the lack of a common definition for the individual categories of RP and their measurement [10–12].

Physical RP appears frequently in the literature on RP use in nursing home settings. Types of physical RP include bedrails, lap belts or physical holds [10]. A study of physical restraint use in people with dementia living in eight European countries reported a mean prevalence of 31.4%, with the lowest found in France (6.1%) and the highest in Spain (83.2%) [13]. A study of 30 nursing homes in Germany found a cluster-adjusted prevalence of residents with at least one physical RP of 26.2% (95% CI 21.3–31.1); bedrails were the most common type of physical RP, used for 24.5% of residents (95% CI 19.5–29.5) [14].

Chemical restraint is more difficult to measure because the administration of a psychotropic drug (e.g. an antipsychotic) does not necessarily equate to it being used as a restraint. Studies have reported the prevalence of use of drugs associated with chemical restraint. The pooled percentage of antipsychotic use in Western European nursing homes was 27% (95% CI 27–28) of residents; in the same study the pooled percentage of antidepressant use was estimated at 40% (95% CI 40–41) [15]. The authors of a Finnish study found a prevalence of regular psychotropic medication use in nursing homes of 60.9% in 2017, having fallen from 81.3% in 2003. Among the classes of psychotropic drugs used in 2017 were antipsychotics (32.7%), antidepressants (32.7%), anxiolytics (14.4%) and hypnotics (6.1%) [16].

Previous research has focused largely on physical and chemical RP use in nursing homes and, consequently, there is a lack of research into other forms of RP such as environmental, social, psychosocial or psychological. Environmental RP — which sometimes encompasses seclusion — usually refers to limiting a person’s movement by means of the surrounding environment (e.g. locked doors or limiting access to a wanted item) [8]. Social, psychosocial or psychological RP (sometimes referred to collectively as ‘informal restraint’) are more nebulous and difficult to accurately define. Examples of these type of RP include diversion, persuasion, white lies or threats [17].

Better understanding of all RP types used would benefit reduction efforts. Thus, we aimed to describe the incidence and type of all reported RP used in Irish nursing homes.

## Research design and methods

### Study design

We conducted a cross-sectional analysis of RP notifications from nursing homes received by the regulator in Ireland in 2020, reflecting the reporting period November-2019 to October-2020, inclusive.

### Population

During the course of 2020 there were 608 nursing homes operating in Ireland, providing 32,091 beds [18]. All nursing homes in Ireland report to the regulator and are thus included in the study.

### Data

We used the Database of Statutory Notifications from Social Care in Ireland for our analysis (HIQA LENS Project: Database of Statutory Notifications from Social Care in Ireland (Internal Version), unpublished) [19]. In addition, data on nursing homes, including their occupancy levels (which are submitted on the first day of January, May and September of each year), were obtained from the regulator’s IT system. The data were pseudonymised by creating new IDs for each nursing home to replace the existing name and code for the nursing home prior to being released to the researchers.

It is a regulatory requirement that nursing homes report “any occasion where restraint was used”, quarterly [20]. The person in charge of the nursing home (typically designated as a Director of Nursing) is legally responsible for submitting such notifications [20]. While a written report is an acceptable means of notification, the vast majority of notifications are submitted via on online portal with pre-defined data fields (HIQA LENS Project: Database of Statutory Notifications from Social Care in Ireland (Internal Version), unpublished). All RP notifications received from nursing homes in 2020 (*n* = 1938) were extracted from the database for analysis using MS Excel [21] and R [22]. There were four categories of RP available to services when submitting a notification: physical, environmental, chemical and ‘other’. Services chose the most appropriate category and, where applicable, RP type. Physical and environmental RP had pre-defined types available for selection: (Physical: bed bumpers, bedrails, chair, lap belt, lap tray/table, other; Environmental: door lock, seclusion, window lock, other). Chemical and ‘other’ RP had no such pre-defined types available.

Each notification can contain up to eight reports of use of RP, we disaggregated these using a MS Excel VBA Macro, creating 6043 notifications. For each notification, service providers state category, type (where applicable), frequency of use, number of residents affected and other details (as free text). We then further disaggregated so that each notification referred to an individual person. In the case of physical and environmental RP, disaggregation was achieved by replicating the notification in the database according to the number of residents affected. This allows for a more accurate estimate of incidence as the data reflects all of the people to which an RP was applied. Notifications with no data in this variable were removed (*n* = 2; 1 physical, 1 environmental). This produced 26,447 physical RP and 37,983 environmental RP notifications. Chemical and ‘other’ RP notifications were disaggregated manually in conjunction with the creation of types for each, as outlined below.

Chemical RP notifications were isolated (*n* = 713). A notification was replicated if it referred to more than one person. Where more than one drug was listed for a person, the notification was replicated to create a unique notification for each drug and a code was produced to link the notifications. Notifications were removed where there were no data in both frequency and number of residents variables (*n* = 7). This resulted in 4103 notifications of chemical RP.

The drug names listed in chemical RP notifications were entered into the Health Products Regulatory Authority’s (HPRA) (the Irish drug and medical device regulator) online database by one researcher to obtain the Anatomical Therapeutic Chemical (ATC) Code [23] for the drug [24]. A second researcher independently repeated the task on 500 (12.2%) notifications, to ensure agreement (a sample of 10%, rounded up to the nearest hundred). The following data were added to each notification: active ingredient, ATC Class 1 and ATC Class 2. Cases where no drug was named were coded ‘Drug not specified’ (*n* = 3569).

RP notifications of type ‘other’ were isolated (*n* = 282). Notifications were removed where there were no data in both frequency and number of residents variables (*n* = 12). The remainder were manually processed by one researcher, producing individual notifications for each person (*n* = 2210). Notifications were sorted and coded to reflect the free text description provided. A second researcher independently coded 300 (13.6%) notifications to ensure agreement (a sample of 10%, rounded up to the nearest 100). The codes were collapsed into themes by one author using an inductive approach (Supplementary File 1).

**Fig. 1:**
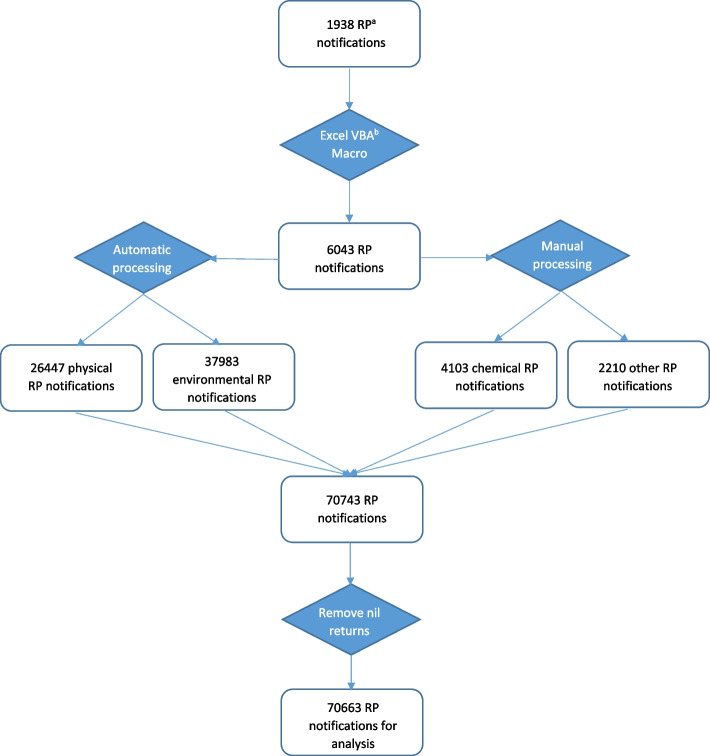
Flowchart describing the processing of notifications prior to analysis. ^a^Restrictive practices. ^b^Visual basic for applications

After processing, each of the four categories combined to produce a total of 70,743 notifications. Data were screened to remove nil returns (*n* = 80). Nil returns are notifications that state an RP was not used during the quarter. The remainder (*n* = 70,663) were included for analysis (Fig. 1).

Occupancy was calculated as the mean value of the three reports submitted by nursing homes to the regulator. Where there were no occupancy data for a nursing home (*n* = 45) we used the nursing home’s registered bed number as of 31st December 2020.

### Analysis

National annual frequency of use of RP was estimated for all categories of RP, and by category and type. The percent contribution of RP types to category, and of category to all RP, was calculated. National incidence of reported RP use was calculated as frequency/occupancy and expressed per 1000 residents, for all facilities operating in 2020 and separately for facilities that reported using at least one RP in the year. Incidence was additionally calculated for all categories of RP and by category and type.

The number of occasions the same resident was administered more than one drug in a single notification was calculated, along with the number of drugs administered to an individual. This was done in order to identify how many individuals were reported to have received more than one drug as a chemical RP during the reporting period. An individual resident may feature more than once among such instances as they may be involved in a notification in more than one quarterly reporting period.

A post-hoc analysis of percent contribution of RP types to the ‘other’ category was carried out using data from quarters three and four (May to October 2020) to account for the introduction of COVID-19 protective measures in nursing homes, some of which were reported as RPs under the ‘other’ category.

Nursing homes that reported at least one RP use in the year, both for any RP use and individual categories of RP use, were expressed as a number and percentage of all nursing homes. Mean quarterly incidence of use, at nursing home level, was estimated as the mean of quarterly reported incidence per resident, for all categories and by category, for these nursing homes. Mean, median, SD, IQR and coefficient of variance was calculated. Boxplots illustrating the distribution of mean incidence for each category of RP were created. In addition, boxplots illustrating the distribution of mean incidence for individual categories of RP, with nursing homes reporting zero use removed, were created.

## Results

Six hundred eight nursing homes were operational during the course of the 12 month period, November-2019 to October-2020 inclusive, with national occupancy of 28,664 (which was calculated as the sum of each nursing home’s mean occupancy). There were 70,663 reported uses of RP over the 12-month period, which was equivalent to 2465.1 per 1000 residents in all nursing homes, and 2848.9 per 1000 residents in nursing homes that reported using RP (Table 1). Environmental was the most frequently reported category of RP (*n* = 37,967; 1324.5 per 1000 residents). Physical was the second highest reported category (*n* = 26,441; 922.5 per 1000 residents). The third most-frequently reported category was chemical (*n* = 4048; 141.1 per 1000 residents). ‘Other’ was the least frequently reported category (*n* = 2207; 77.0 per 1000 residents).

**Table 1:** Types of restrictive practices used in nursing homes in Ireland in 2020

Category	Type	Frequency	% Contribution to Category	Incidence (All NH^a^)/Per 1000 Residents	Incidence (NH^a^ that reported RP^b^)/Per 1000 Residents
Physical	Bedrails	16,843	63.7	587.6	679.1
	Bed bumpers	3508	13.3	122.4	141.4
	Other - physical	3284	12.4	114.6	132.4
	Lap belt	1772	6.7	61.8	71.4
	Chair	889	3.4	31.0	35.8
	Lap tray / table	145	0.5	5.1	5.8
	**Total physical**	**26,441**	**100**	**922.5**	**1065.9**
Environmental	Door lock	29,296	77.1	1022	1181.2
	Window lock	4906	12.9	171.2	197.8
	Other - environmental	3477	9.2	121.3	140.2
	Seclusion	288	0.8	10.0	11.6
	**Total environmental**	**37,967**	**100**	**1324.5**	**1530.8**
Chemical	Drug not specified	3449	85.2	120.3	139.1
	Antipsychotics	294	7.2	10.3	11.9
	Anxiolytics	233	5.8	8.1	9.4
	Hypnotics and sedatives	48	1.2	1.7	1.9
	Antiepileptics	12	0.3	0.4	0.5
	Antidepressants	7	0.2	0.2	0.3
	Opioids	3	0.1	0.1	0.1
	Antidementia drugs	1	0.0	0.0	0.0
	Dopaminergic agents	1	0.0	0.0	0.0
	**Total chemical**	**4048**	**100**	**141.1**	**163.2**
Other	Privacy	722	32.7	25.2	29.1
	Covid-19 protective measures	634	28.7	22.1	25.6
	Liberty & autonomy	278	12.6	9.7	11.2
	Physical restraint - other	235	10.6	8.2	9.5
	Environmental restraint - other	136	6.1	4.7	5.5
	Mobility	82	3.7	2.9	3.3
	Safety	78	3.5	2.7	3.1
	Restraint not specified	40	1.8	1.4	1.6
	Chemical restraint - other	2	0.1	0.1	0.1
	**Total other**	**2207**	**100**	**77**	**89**
	**Total any restrictive practice**	**70,663**	**100**	**2465.1**	**2848.9**

^a^Nursing homes

^b^Restrictive practices

In the physical RP category, bedrails were the most frequently reported (*n* = 16,843; 587.6 per 1000 residents), contributing almost two-thirds to this category (63.7%).

For environmental RP, the most frequently reported RP type was door lock (*n* = 29,296; 1022.0 per 1000 residents). Second most frequent was window lock (*n* = 4906; 171.2 per 1000 residents). When combined, door lock and window lock accounted for the majority of types within this category (90.0%).

Under chemical RP, no drug was specified in the majority of notifications (*n* = 3449; 85.2%). Where a drug was specified, the majority (96.0%) were: antipsychotics (*n* = 294; 10.3 per 1000 residents); anxiolytics (*n* = 233; 8.1 per 1000 residents); and hypnotics and sedatives (*n* = 48; 1.7 per 1000 residents). There were 37 instances of a resident being administered multiple drugs (2 drugs: *n* = 32; 3 drugs: *n* = 5) out of a total of 4048 notifications for chemical RP. An individual resident may feature more than once among the 37 instances as they may be involved in a notification in more than one quarterly reporting period.

In the ‘other’ RP category, the theme of privacy was the most frequently reported type (*n* = 722; 32.7%). Codes within this theme described restrictions such as motion alarms (devices that notify staff if a person is mobilising) and listening devices (Supplementary File 1). Second most frequently reported was the theme of Covid-19 protective measures (*n* = 634; 28.7%). These were typically restrictions that were specific to measures introduced on public health grounds such as restrictions on visitors to the nursing home or limits on accessing the community due to the risk of contracting Covid-19. The theme of liberty & autonomy was the third most frequently reported type (*n* = 278; 12.6%). This theme included codes such as access to cigarettes or alcohol and alarm bracelets (devices worn on a person’s body which notify staff if the person passes a certain location e.g. an exit door). For quarters 3 and 4 only, Covid-19 was the most frequently reported RP type (*n* = 529, 39.4%) in the ‘other’ category. Privacy was second most frequent (*n* = 357, 26.6%); liberty & autonomy was third most frequent (*n* = 151, 11.3%) (Table 2).

**Table 2:** Frequency and % contribution of types of ‘other’ reported restrictive practices in nursing homes in Ireland

Type	Frequency	% Contribution to Category
COVID-19 protective measures	529	39.4
Privacy	357	26.6
Liberty & autonomy	151	11.3
Environmental restraint – other	108	8.1
Physical restraint – other	68	5.1
Safety	51	3.8
Mobility	38	2.8
Restraint not specified	38	2.8
Chemical restraint – other	1	0.1
**Total**	**1341**	**100**

These data are for quarter three and quarter four of 2020 only (6 months), to account for impact of the introduction of Covid-19 related public health guidance

Five hundred fifty nursing homes (90.5%) reported using at least one RP in the 12-month period, meaning 58 (9.5%) nursing homes reported using no RP in the 12-month period. Most nursing homes (*n* = 527; 86.7%) reported using at least one physical RP. This was followed by environmental (*n* = 298; 49%); chemical (*n* = 233; 38.3%); and ‘other’ (*n* = 109; 17.9%).

**Fig. 2:**
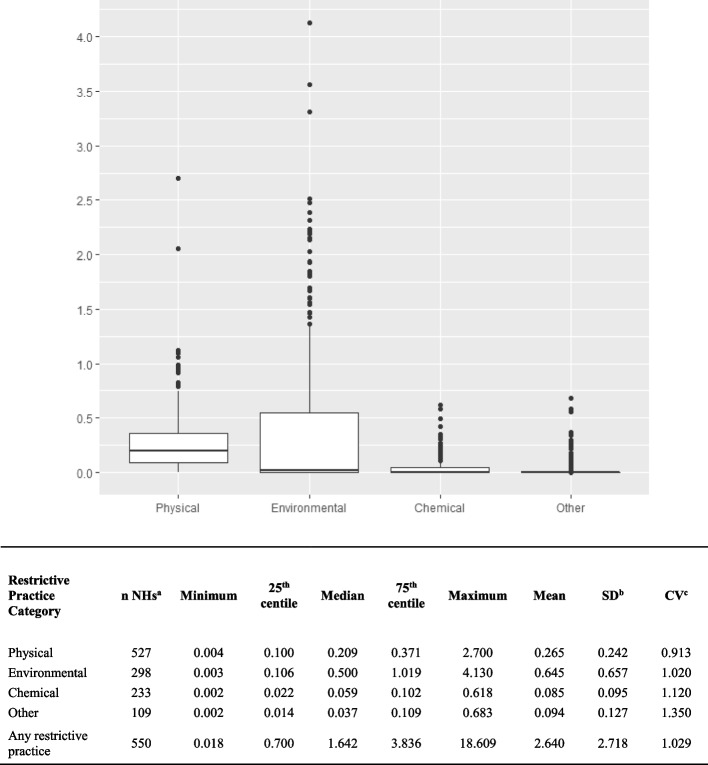
Incidence of use of restrictive practices per resident in nursing homes in Ireland in 2020. Descriptive summary of mean quarterly incidence of use per resident of reported restrictive practice by category, in nursing homes in Ireland reporting restrictive practice use, from statutory notifications received in 2020. (Outliers were identified as facilities with incidence outside of 1.5 times the IQR)

There was high variance (CV > 1.00) for each category except physical, in mean quarterly incidence of RP use in individual nursing homes that reported using RP (Fig. 2). The mean quarterly incidence for any RP use in nursing homes that reported using RP was median 1.642 (IQR: 0.018 to 18.608) per resident.

The boxplot in Fig. 2 illustrates the distribution of mean incidence on the same scale for each category of RP in nursing homes that reported using any RP. A second boxplot illustrates the distribution of mean incidence for each category of RP, each on their own respective scale, with nursing homes reporting zero use removed (Supplementary File 2). For both boxplots, outliers are represented by dots and have a mean incidence outside of 1.5 times the inter-quartile range.

## Discussion and implications

In this large cross-sectional study, 90.5% of nursing homes submitted at least one notification for any RP use in the year 2020. 70,663 instances of RP use (2465.1 per 1000 residents) were reported. There was large variance in the frequency of reported use across nursing homes. A small number of nursing homes reported a high mean incidence of use when compared with all nursing homes. These nursing homes are represented as outliers in the boxplot (Fig. 2).

A wide range of RP types were reported. The most frequently reported category of RP was environmental, despite less than 50% of nursing homes reporting using environmental RP. This category did not feature often in the literature suggesting it is an under-studied phenomenon. Moreover, ‘other’ RP (such as social, psychosocial or psychological) contributed notably to overall use of RP in Irish nursing homes. It may be the case that the lack of a clear definition for these types of ‘informal’ RP leads to grey areas and differences in understanding of the phenomenon across jurisdictions [17]. We did not identify other studies that investigated the incidence or frequency of use of ‘other’ RP, suggesting this category is also under-studied.

To the best of our knowledge this study is the first to describe environmental, physical, chemical and ‘other’ RP use in nursing homes from a national perspective. Cross-comparison of incidence of RP in nursing homes was complicated by the differing terminology employed across jurisdictions. For example, the database used in the herein study included bedrails as a type of physical RP whereas in other studies it was defined as a mechanical RP [25, 26]. As such, it is not possible to determine whether the incidence of RP reported in this study is low or high compared with similar settings in other countries. This serves to highlight the importance of agreed definitions for RP as well as effective measurement which would allow for cross-comparison.

Similar to Meyer et al. [14], we found that bed rails were the most common form of physical RP used in a nursing home setting (albeit that Meyer et al. use the terms physical and mechanical interchangeably). We also found that use of tables or belts as a form of physical RP was relatively low in comparison to bed rails, as in Meyer et al. [14] Similarly, bed rails were the most common form of physical RP in a systematic review published in 2021 [27]. However, the authors of that review found that prevalence rates for physical and chemical RP were broadly equivalent to each other which is in marked contrast to our findings where the incidence of physical RP per 1000 residents was 6.5 times greater than that of chemical RP. The authors drew attention to the large heterogeneity in the estimates and differences in terms of measurement and geographic location of the study. These factors may also explain the incongruence between our findings and Lee et al. [27]

Our findings in respect of chemical RP show that the vast majority of notifications (85.2%) did not include the drug name. Where the drug names were specified, the first, second and third most frequently reported were antipsychotics (49%), anxiolytics (39%) and hypnotics & sedatives (8%). There is some agreement in our findings and those of Lee et al. in respect of the drugs most frequently used for chemical RP [27]. In that study, the authors ranked the types of drugs most frequently used for chemical restraints and found that benzodiazepines were highest with a pooled prevalence of 42%; followed by antipsychotics (38%); antidepressants (37%), neuroleptics (29%); antiepileptics (19%); anxiolytics (13%) and hypnotics (1%). Benzodiazepines fall under the hypnotics and sedatives classification which featured third in our study in terms of drugs most often reported.

Covid-19 public health restrictions were introduced in Ireland in March 2020 [28] and subsequently fluctuated in implementation throughout the remainder of the year. As such, it is difficult to estimate the true contribution of RP related to Covid-19 in the year. Our post-hoc analysis of the two quarters subsequent to the emergence of Covid-19 in Ireland showed Covid-19 as the most frequently-reported RP type under ‘other’ (39.4% contribution to category). The order of frequency of use of RP types in the ‘other’ category remained largely unchanged when the Covid-19 type was excluded.

### Policy and practice implications

Improved understanding of all RP forms will allow for greater oversight and development of interventions to reduce their use. In policy implications, there is a need to standardise the definitions for all forms of RP to support improved reporting and to allow comparison across countries, as demonstrated from the range of RPs identified in this study and the disparity in the literature. In addition, a greater focus on RP other than physical and chemical is warranted. The more subtle forms of ‘other or ‘informal’ RP may be under-reported due to lack of regulatory or legislative requirement to do so, and possibly lack of knowledge of service providers that various acts could constitute a RP. This potential under-reporting should not be taken as an indication that these forms of RP are a less severe infringement of the rights of older people living in nursing homes. Indeed, these RP have the potential to be more insidious and warrant greater vigilance among providers and professionals to detect and eliminate – or limit – their use.

### Strengths and limitations

Previous literature on RPs in nursing homes has primarily focused on physical and chemical RP. As such, a strength of this study is the use of data on all reported RP forms used in Irish nursing homes. Moreover, the data represents a national view of RP use as it includes all Irish nursing homes. A further strength is found in the inclusion of the category of ‘other’ RP. This did not limit the RP types that could be reported and thus facilitated analysis not limited by a priori defined lists.

Limitations of the study warrant consideration. Each notification represented one instance of RP use per resident and did not account for duration of use. Therefore, estimates of incidence could be considered underestimates. However, all notification types and categories were treated the same thus the reporting is comparable across nursing home and type and the % contributions remain valid. Furthermore, RP notifications continue to be collected in this manner, meaning the incidence as reported herein will be comparable across time for future analyses.

Data were self-reported and as such there was a possibility of reporting bias. However, under-reporting is likely to have been minimal because these notifications are a regulatory requirement [20]. Nevertheless, it cannot be discounted that there may have been RP used that were not recognised as restrictive and thus were not reported. The presence of nil returns in the dataset also suggests under-reporting is minimal.

It is possible that there is over-reporting in these data. For example, some notifications reported drugs that would likely not meet the definition for chemical RP (e.g. opioids; dopaminergic agents; antidementia drugs). We retained this small number of notifications in our analysis for completeness.

More than 85% of chemical RP notifications had no drug listed. This missingness likely impacts on the hierarchy of drugs reported.

Data on the occupancy of nursing homes is only submitted to the regulator on three occasions per year. While occupancy will fluctuate much more frequently than this, there were no other measures available to reflect the number of people living in a nursing home at a particular point in time. However, we are satisfied that mean occupancy is a more appropriate figure than the number of registered beds for the purposes of calculating incidence.

There was some overlap across the four categories of RP in these data as evidenced by the reporting of environmental, physical and chemical RP under the category of ‘other’. We retained use of RP as reported, as it highlights the need for improving definitions and understanding of RPs generally. If we had reclassified this small number of notifications, it would have marginally changed the frequency and incidence reported but it would not have impacted on the rankings of contribution of the categories to total RP or our overall conclusions.

## Conclusion

RPs are commonly used in nursing homes in Ireland and only 9.5% reported being restraint-free in the 12-month period. A wide variety of RP types were reported. Environmental and ‘other’ (social, psychosocial, psychological) RP contributed notably to total RP use and warrant attention alongside the traditional focus on physical and chemical RP.

Our findings make the case for further investigation into forms of RP used in nursing homes and for standardised definitions which are comprehensive for all RP. The findings can inform policy and practice relating to the use, monitoring and reduction of RP which may ultimately improve human rights for older people in nursing home settings.

## Supplementary Information

**Additional file 1.**

**Additional file 2.**

## Data Availability

The database used for this study is not publicly available as it contains regulatory data about the performance of nursing homes in Ireland. However, the data can be made available from the corresponding author on reasonable request and under a data sharing agreement. In accordance with the European Union’s Open Data Directive [29], a public version of the database, with sensitive data relating to individual services removed and/or de-sensitised, is available at the following webpage: https://www.hiqa.ie/areas-we-work/Database-of-Statutory-Notifications
