# Supplementary material for: Incidence and type of restrictive practice use in nursing homes in Ireland
Source: BMC Geriatr. 2022 Oct 15;22:802. doi: 10.1186/s12877-022-03450-4 (PMC9569185; doi:10.1186/s12877-022-03450-4)

**Supplementary File 2** – Boxplot illustrating distribution of mean incidence for each category of RP, excluding centres reporting zero use


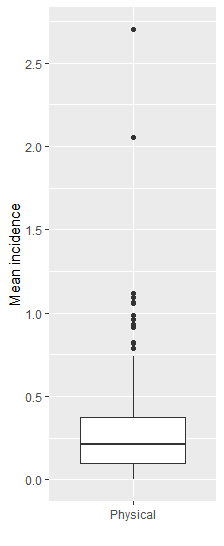

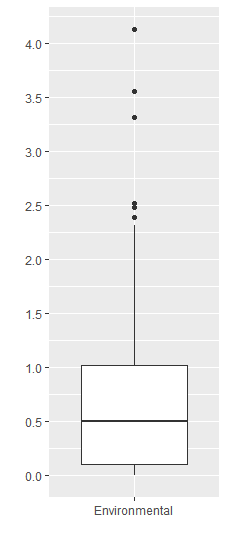

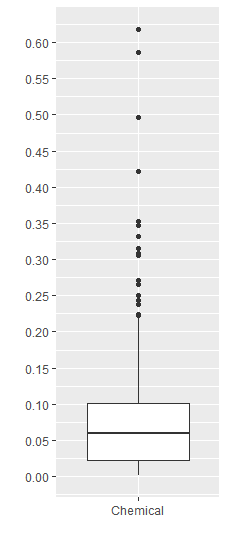

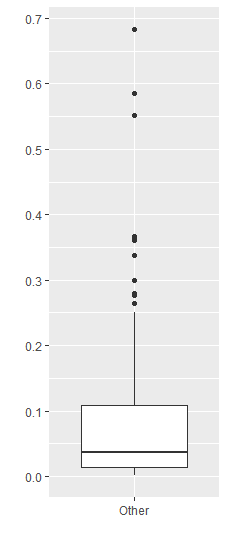

Supplement: Supplementary file 2 — Additional file 2. [file 12877_2022_3450_MOESM2_ESM.docx]
