# Supplementary material for: Incidence and type of restrictive practice use in nursing homes in Ireland
Source: BMC Geriatr. 2022 Oct 15;22:802. doi: 10.1186/s12877-022-03450-4 (PMC9569185; doi:10.1186/s12877-022-03450-4)

Additional File 1 Coding tree for "other" reported restrictive practice use in nursing homes in Ireland, in statutory notifications spanning November 2019 to October 2020.

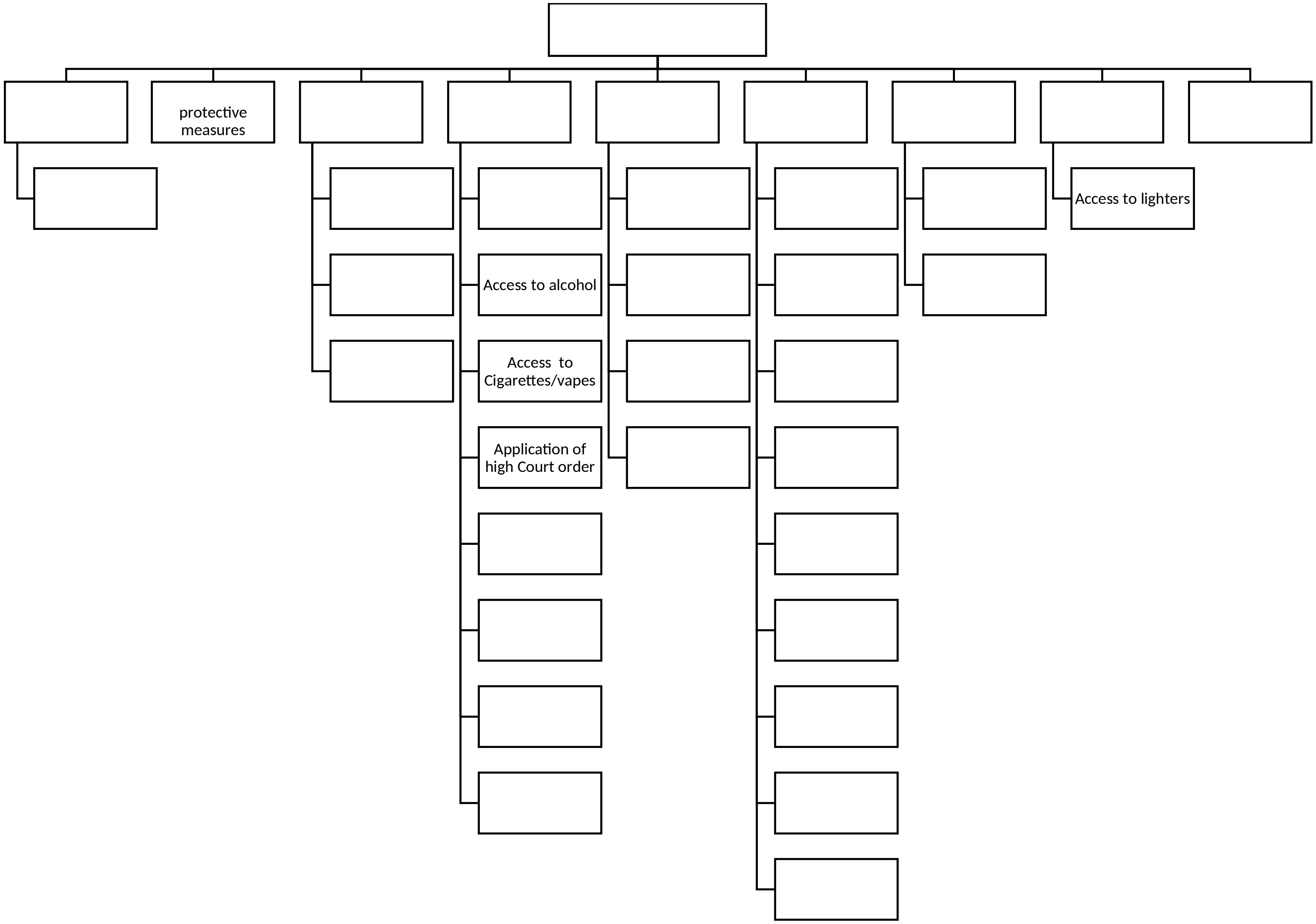

Supplement: Supplementary file 2 [file 12877_2022_3450_MOESM1_ESM.docx]
